# Auto-MuRCiS: a streamlined software package for analysis of multiplex, randomized CRISPR interference sequencing data

**DOI:** 10.1128/mra.00356-24

**Published:** 2024-07-31

**Authors:** Kevin S. Myers, Michael Place, Audrey P. Gasch, Daniel R. Noguera, Timothy J. Donohue

**Affiliations:** 1 Wisconsin Energy Institute and Great Lakes Bioenergy Research Center, University of Wisconsin-Madison, Madison, Wisconsin, USA; 2 Laboratory of Genetics and Center for Genomic Science Innovation, University of Wisconsin-Madison, Madison, Wisconsin, USA; 3 Department of Civil and Environmental Engineering, University of Wisconsin-Madison, Madison, Wisconsin, USA; 4 Department of Bacteriology, University of Wisconsin-Madison, Madison, Wisconsin, USA; University of Maryland School of Medicine, Baltimore, Maryland, USA

**Keywords:** CRISPRi, docker, python, pipeline, computation

## Abstract

Multiplex, randomized CRISPR interference sequencing (MuRCiS) allows for the simultaneous identification of multiple gene knockouts that together influence microbial processes. Here, we report on an updated analysis tool called Auto-MuRCiS that utilizes Docker to make the analysis of these data rapid and more user-friendly.

## ANNOUNCEMENT

**Fig 1:**
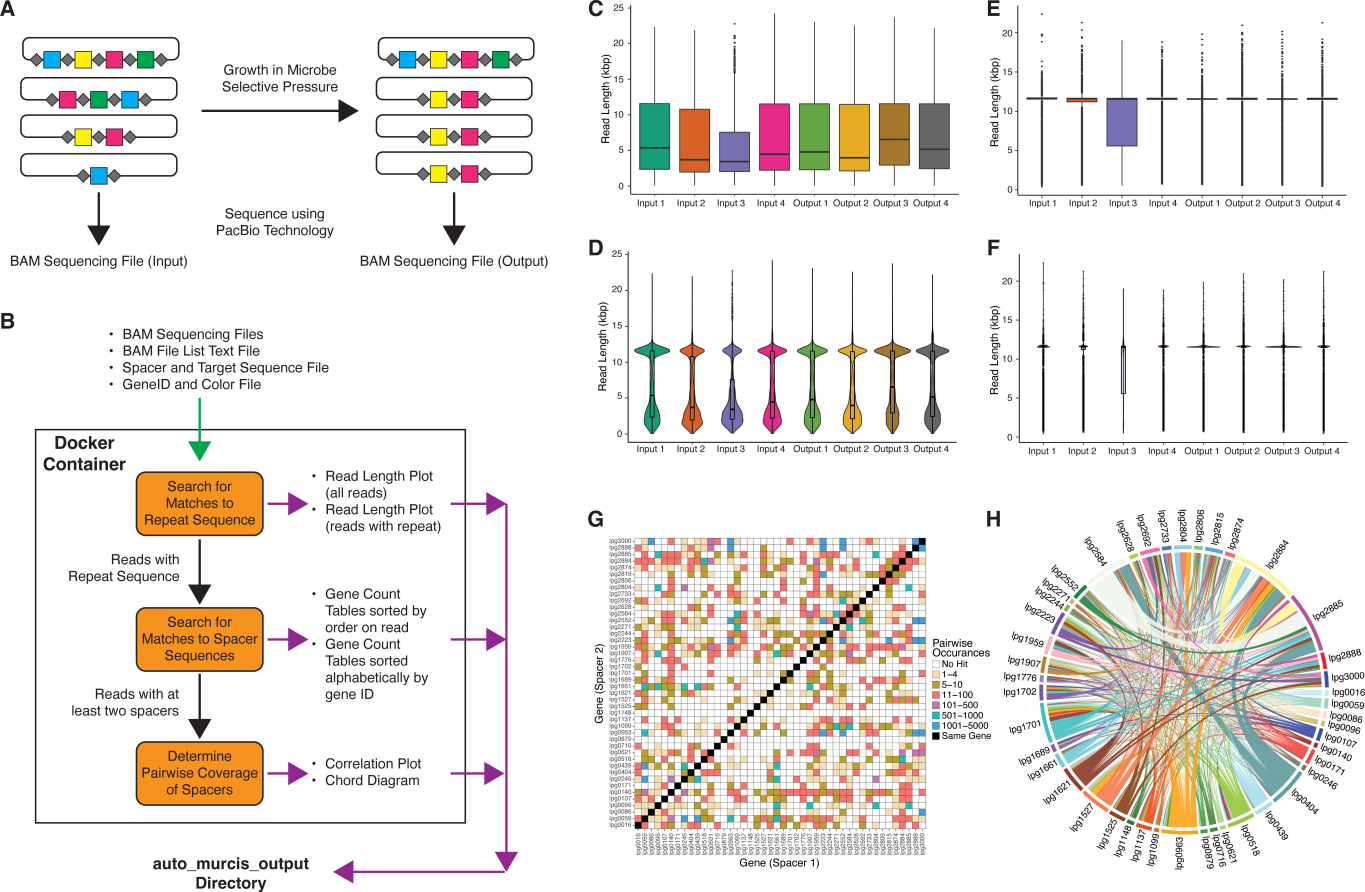
The Auto-MuRCiS pipeline and output. (A) Overview of MuRCiS procedure. Random combinations of CRISPRi spacers that target specific genes (colored squares) are separated by repeats (gray diamonds) on a plasmid. The plasmid is transformed into a microbial library and grown in defined conditions, and the plasmids in the input and output are isolated and sequenced using long-read sequencing. (B) A flow chart illustrating the Auto-MuRCiS pipeline. The pipeline is run in a Docker container and the single Bash script copies files into and out of the Docker container. The input files are shown at the top with green arrows, and the general steps of the pipeline are in orange boxes. The output files are listed with purple arrows, and specific files used in the steps of the pipeline are listed alongside the black arrows. All analyses can be run from a single script (murcs_script.py) if Docker is not available. (C and D) Box plots (C) and violin plots (D) for all sequenced reads. (E and F) Box plots (E) and violin plots (F) for reads with matches to the repeat sequence, note the smaller distribution compared to C and D plots. (G) Correlation plot showing pairwise instances of all possible pairs of spacers. Squares are colored based on number of times each spacer pair is identified. (H) Chord diagram showing a number of times each spacer pair appears together in a single read, with colors defined by the user in the Color File.

CRISPR interference (CRISPRi) can be used to inhibit or reduce the expression of specific genes, making it ideal to study gene function and its influence on phenotype (1). Multiplex, randomized CRISPRi sequencing (MuRCiS) can silence multiple, random sets of genes using defined and unique spacers in a single experiment (2). The MuRCiS procedure can be used to interrogate combinatorial effects of multi-gene inhibition in microbes, allowing researchers to study the impact of knocking down multiple genes (Fig. 1A) (2). However, analysis of these data as reported previously required comprehensive knowledge of bioinformatics and running many separate scripts, limiting its scope and utility. Here, we present Auto-MuRCiS, an open-source analysis pipeline to reproducibly process and analyze data from MuRCiS experiments (Fig. 1B).

Auto-MuRCiS utilizes a Docker image that maintains a consistent and reproducible environment in which to run the associated scripts (3). A single Bash script command is used and requires only three inputs to run using a single line of code: (i) a text file with the Binary Alignment Map (BAM) sequencing files to process; (ii) a two-column, tab delimited, text file indicating the sequence of the repeat and the various spacer sequences used in the experiment where the first column indicates the name of the sequence (“repeat” or the spacer ID) and the second column indicates the corresponding 5′ to 3′ DNA sequence; (iii) a two column, comma delimited, text file indicating the preferred colors to use for each gene in the Chord diagram plots generated by Auto-MuRCiS. Example input files can be found in the Example directory on GitHub. Auto-MuRCiS is designed to work with CCS BAM files from Pacific Biosciences (4). Modification would allow it to utilize other long-read sequencing methods, such as Oxford Nanopore Technologies (5). On a standard desktop or laptop computer (e.g., Intel Core i7 with 32 GB RAM) Auto-MuRCiS can process an experiment in ~30 minutes.

Auto-MuRCiS processes the data to report the lengths of reads with and without repeat sequences to visualize the differences as a control. The results are both written to text files and plotted as box and violin plots (Fig. 1C through F). The number of exact matches of spacer sequences provided for each gene is recorded for reads containing repeat sequences. Auto-MuRCiS will export the gene/spacer count values from each experiment to text files. Auto-MuRCiS will report on the pairwise distribution of events, which is how often each pair of spacers appear together in a read, regardless of the other spacers present in the read. Auto-MuRCiS will produce two publication-ready graphics: a correlation plot and a Chord diagram (Fig. 1G and H). The correlation plot can be used to gain an understanding of the overall distribution of the spacer pairs, while the Chord diagram can provide an easy way to view which pairs are most common in the data (using the user-provided color information). All output data are contained in a single directory that is copied out of the Docker container. Auto-MuRCiS will allow researchers to more easily adopt the powerful MuRCiS technology and address widespread biological questions.

## Data Availability

The Auto_MuRCiS scripts are available on GitHub: https://github.com/GLBRC/auto_murcis. The Auto_MuRCiS Docker image is available on the Docker Hub (login required): https://hub.docker.com/repository/docker/kevinmyers/auto_murcis/general.
